# Management of Transverse Presentations of the Fœtus

**Published:** 1857-01

**Authors:** John Hardin

**Affiliations:** Professor of Obstetrics in the Kentucky School of Medicine at Louisville


Art. II.—*A Paper on the Management of Transverse Presentations of the Fœtus.* Read before the Louisville Medical Club. By Jno. Hardin, M.D., Professor of Obstetrics in the Kentucky School of Medicine.

In the following remarks, it will not be necessary for me to notice the prevailing mode of practice in transverse presentations of the fœtus, further than briefly to state it, so as to present fairly, in contrast with it, other methods, which, it seems to me, have received too little attention.

We are instructed, by nearly all the obstetric authorities current in this country, that, in the treatment of arm and shoulder presentations, we should introduce the hand into the uterus, and carry it onward, until it comes in contact with the foot, or knees of the fœtus; and, having seized one or both feet, or one or both knees, we are to accomplish the version, and deliver, as if the case had been originally a foot presentation.

Some of our teachers think it very important that both feet should be brought down when it can be done; while others prefer to bring down one only; even in those cases where it would be perfectly easy to seize both. The former prefer both, because the child is thus brought more nearly to the shape of a wedge, and, therefore, the nates more easily pass the os uteri; while the latter regard delivery by traction on one limb as preferable, because the bulk of the nates being increased by the addition of one thigh folded upon the abdomen, more perfectly dilates the parts through which it must pass; and thereby renders the transmission of the head more easy and diminishes the risk to the child from compression of the funis. In those cases in which the abdomen of the child looks anteriorly, they prefer delivery by traction on one limb, for the further reason, that by this method, rotation is certainly accomplished, so as to bring the dorsal aspect of the child to the abdomen of the mother,—a point of prime importance, when the head comes to pass through the pelvis.

Dr. Simpson has suggested a mode of procedure, partly in accordance with the last-mentioned method, involving a material improvement; this relates to a choice as to the limb which shall be brought down. While he prefers, for the reasons already assigned, to bring down but one limb, he thinks there is abundant reason for giving the preference to that limb which is the opposite of the presenting shoulder,—thus, if the right shoulder present, he selects the left foot; and if the left shoulder present, then he selects the right. By acting on the leg which is opposite to the presenting shoulder, he accomplishes rotation as well as flexion, and this rotation withdraws the presenting shoulder from its position at the os uteri. Whereas, if the leg corresponding to the presenting shoulder, is brought down, it is accomplished simply by flexion of the body of the child upon itself. This correction of position is not so easy by this method as by the other, where we combine the movement of rotation with flexion.

I hope the Club will indulge me in the relation of a case that I encountered last spring, in conference with Dr. O'Reilly, of this city, in which I was enabled to turn and deliver, without introducing more of my hand than the fingers, into the uterus.

The left hand of the child was protruding from the vulva, the head to the right side, and the back looking forward. The woman was placed upon her back, and brought fully under the influence of chloroform. I introduced the whole of my right hand into the vagina, and placed two fingers in the axilla of the child and applied the left hand to that part of the abdomen of the mother, against which the fœtal head rested; a double movement was then given to the child, by pressing, with my right hand, the shoulder laterally towards the fœtal head, while, at the same time, the left hand, resting low down upon the side of the abdomen, pressed the head upwards towards the fundus of the uterus. By this movement, which was accomplished with great facility and despatch, the pelvic extremity of the ovoid was brought directly over the pelvic aperture, and a foot seized, without the further introduction of the hand into the uterus; no part of the hand, except the fingers, entering that cavity, and my wrist being still surrounded by the vulva. In this case, both mother and child, which latter was large, did well; I may add, however, that I should not have been able to deliver the head in time to save the child, but for the assistance of one blade of the forceps, used as a vectis. Although the manipulation described in this case has not hitherto been used, as far as I know, in any case of podalic version, I do not claim to have originated the method, but only used to accomplish version by the feet, a mode, to which I shall again refer, which has been advised for the accomplishment of cephalic version.

In cases of transverse presentation, where the membranes have been ruptured and the waters discharged, the uterus contracts and is moulded to the form of the ovoid. The long diameter of the child, lying across the abdomen, from ilium to ilium, the greatest diameter of the uterus must be in the same direction; but the presenting shoulder dipping down into the true pelvis, causes the body of the child to be flexed laterally, and thus brings the cephalic and pelvic extremities nearer together. If, under these circumstances, we push the shoulder directly upwards, we shall straighten the body, and thus widen the distance between its two extremities, and by so much increase the lateral diameter of the uterus; and when the force elevating the shoulder is removed, the uterine contractions will immediately force it down to its former position; but if the pressure upon the shoulder is made in a lateral direction, and at the same time, by a hand applied externally, the head is pressed upward toward the scrobiculus cordis, the fœtal and uterine surfaces being curvilinear, glide easily, one upon the other, and the long diameter of the ovoid is made to correspond to the long diameter of the maternal abdomen, the uterus participating in the same change as that of its contents.

It only remains now for me to notice briefly cephalic version, a method as old as the annals of medicine, but one which has nearly fallen into disuse; and it is no wonder that it is so, when we consider the difficulty of its accomplishment, if attempted in accordance with the directions of our teachers.

Some of these tell us that the mode of procedure is to "push the shoulder up out of the way, grasp the head and bring it down to the brim of the pelvis;" a method not to be accomplished either easily by the accoucheur, or with safety to the mother. Others do not allude to it at all, while some mention it only to reprobate it.

More than twenty years ago, Dr. Hodgen, of Campbellsville, Kentucky, and myself, were summoned to a case of arm presentation, which had been under the management of a midwife for eight hours. The waters were discharged, the hand protruded externally, and the labor pains were very strong and propulsive. The woman, who was muscular and plethoric, was placed in the sitting posture on the edge of the bed, her feet resting on chairs, pillows being arranged behind her ready for use at the proper time. I bled her from a large orifice to syncope; as soon as this was induced she was laid down, and before she recovered Dr. Hodgen had rectified the position by returning the arm, pressing the shoulder laterally away from the os uteri, and thus allowing the head to drop into its place. In half an hour the labor was accomplished by the natural efforts.

During my residence in Greensburg, my friend and former partner, Dr. D. P. White, substituted the head for the shoulder, and gave the case up to the natural efforts, which safely finished the delivery.

Dr. S. B. Richardson of this city, informs me that he has converted three shoulder into vertex presentations; and I had hoped to have had a detailed statement of these cases to submit to you, but have been disappointed in my expectations of receiving them.

Dr. M. B. Wright, of Cincinnati, formerly Professor of Obstetrics in the Medical College of Ohio, has published a paper, which, I think, was submitted to the Ohio State Medical Society, containing an interesting detail of seven cases (if my memory is correct), in which the head was substituted for the shoulder; most, if not all, of which cases he saw in consultation. In some of them the membranes had been ruptured for more than twenty-four hours, and, if I recollect aright, in some for twice or thrice so long a time. In some of the cases ineffectual efforts had been made by other physicians, and then by himself, to reach the feet and turn by that method; and in these cases he succeeded in converting the shoulder into a head presentation. I regret exceedingly that I have mislaid his paper, and it is so long since I read it that I cannot give the interesting details as fully as I desire to do. More especially do I regret its loss, as otherwise I should avail myself of the opportunity to transcribe his description of his mode of manipulation; but it is substantially as follows. Having ascertained which shoulder presents, and to which side of the mother the child's head is directed, the hand is introduced into the vagina. If the fœtal head is to the mother's right side, the left hand is to be used; if the head is to her left side, the right hand is to be preferred. The protruding arm is to be returned into the uterus, then the fingers are placed upon the top of the shoulder, and pressure made laterally towards the child's pubis, and at the same time the other hand, applied on the outside of the abdomen, bears the pelvic extremity of the ovoid up towards the fundus uteri; the fœtal surface being convex glides easily over the concave surface of the uterus, and as the shoulder is borne away from the os uteri, the head necessarily falls into and occupies it. By this change in the situation of the child, the shape of the uterus is altered—its vertical instead of its lateral diameter being the longest—and there is no further danger of the head being turned aside, and the shoulder occupying its place.

We are informed by Churchill, that Wigand asserts the practicability of making this change solely by external manipulation; and Velpeau has confirmed the statement by his own experience.

In any of these operations, the great obstacle to success is uterine contraction. Formerly we were taught to use the lancet, tartarized antimony, and opium. Of these, the lancet is much the most efficient, if used to the extent of producing syncope, as in the case I have detailed. If the operation be performed before the woman recovers from this state, there is little or no resistance offered by the uterus; but it may not be safe to induce syncope, for several reasons too obvious to require specification. Opium and tartarized antimony will mitigate, but cannot suspend, the uterine actions. Fortunately, modern investigation has brought to our knowledge a remedy, prompt, efficient, and so nearly safe that we do not hesitate to employ it in preference to all others. This remedy, I need scarcely say, is chloroform, used to the extent of producing as full and complete anæsthesia as would be required for amputating a limb. It holds the uterine action in complete abeyance, so that we may calmly and considerately employ any mode of changing the presentation of the child that we may desire.

From what I have said, you will have perceived that I regard cephalic version as not only practicable, but, under some circumstances, preferable to any other. Dr. Churchill concludes from statistical data, that in podalic version, one mother perishes in every sixteen cases, and one child in rather less than three. The danger to the mother arises from laceration of the uterus, from the shock of the operation, or from inflammation following delivery on account of contusions or other injuries. The danger to the child springs from compression of the umbilical cord, while the chest and head are passing through the pelvis.

I conclude, then, that cephalic version should be preferred in those cases where the pelvis is of average size, where the uterus is still capable of vigorous propulsive action, and where there is no necessity for hastening delivery.

On the contrary, podalic version is preferable where the uterus has become exhausted, so that the natural efforts cannot be relied on to accomplish the delivery; where the brim of the pelvis is contracted to such an extent as to offer a serious obstacle to the transmission of the head; where puerperal convulsions, dangerous hemorrhage, or any other circumstance indicating the necessity for speedy delivery, occur.

